# Machine learning combine with nomogram to guide the establishment of endoscopic assistant system for gasless transaxillary endoscopic thyroidectomy

**DOI:** 10.1080/07853890.2025.2537354

**Published:** 2025-07-25

**Authors:** Linjie Ma, Yuqiu Zhou, Chao Li, Xu Wang, Tong Liu

**Affiliations:** Department of Thyroid Oral and Maxillofacial Surgery Sichuan Cancer Hospital, Sichuan Clinical Research Center for Cancer, Sichuan Cancer Hospital & Institute, Sichuan Cancer Center, University of Electronic Science and Technology of China, Chengdu, China

**Keywords:** Endoscopic assistants, gasless transaxillary endoscopic thyroidectomy, CUSUM learning curve, machine learning, nomograph

## Abstract

**Objective:**

To explore the influence related factors of endoscopic assistant in gasless transaxillary endoscopic thyroidectomy by using machine learning and nomogram, and construct an endoscopic assistant system.

**Methods:**

A skilled endoscopic assistant(Group A, *n* = 50)and an unskilled endoscopic assistant(Group B, *n* = 50)were randomly included in participating operation, CUSUM was calculated and learning curve was constructed. Age and other factors were included to study CET, TRT, TST, LWT and CUSUM. Univariate and multivariate analysis were conducted to find out the relevant factors affecting the surgical process. Nomogram and machine learning were constructed to find out the influence of relevant factors on the surgical process.

**Result:**

The learning curve coefficient of goodness of fit R^2^=0.807. The cases in the learning stage of skilled assistant and unskilled assistant were 20 and 28. In mastery stage, the surgical time of skilled assistant had less fluctuation than that of unskilled assistant. There were statistical significance in CET(*p* = 0.001), TST(*p* = 0.001), LWT(*p* = 0.002), CUSUM(*p* = 0.019). In multivariate analysis, CET(*p* = 0.004), LWT(*p* = 0.013), CUSUM(*p* = 0.025), TRT(*p* = 0.018) showed statistical significance. Nomogram was successfully constructed based on the relevant factors explored by the multivariate analysis, and the influence of each relevant factors were explored by machine learning. The system was constructed through preclinical training, preoperative preparation, and share experience from the perspective of the endoscopic assistant according to the procedure process.

**Conclusion:**

It is necessary to train endoscopic assistant to build an endoscopic assistant system, and improve the surgical process by shortening CET, TRT and reduce LWT times. The importance of experience accumulation to improve the efficiency of surgery should be emphasized.

## Introduction

Currently, there are two main types of endoscopic thyroidectomy approaches. The first is the approach that requires the establishment of a cavity, for example, through the axillary areola. The second is the approach that utilizes the natural cavity of the human body, mainly including the oral approach [1–6] Regardless of the endoscopic approach, compared to thoracoscopy and laparoscopic surgery, the utilization of the thyroid endoscopic surgery cavity is relatively difficult, even after the cavity is established, it still faces the limited space problem. Our center proposed a six-step method for cavity establishment in gasless transaxillary endoscopic thyroidectomy [7]. Compared to thoracoscopy and laparoscopy, there are fewer reports on the experience summary of the endoscopic thyroidectomy assistant, and the establishment of assistant system.

At present, There were rare researches on the improvement of the efficacy of the endoscopic assistant in gasless transaxillary endoscopic thyroidectomy, and the effect of the endoscopic assistant on the surgical process is unknown. The multilayer perceptron (MLP) [8,9], as the cornerstone of machine learning [10–12], has the function of looking for potential linearity and nonlinearity, and explores the influence of related factors on the outcome by constructing neural networks. By constructing an endoscopic assistant learning curve, we searched for the related factors to the influence of the endoscopic assistant on the surgical process, explored the effect of these factors by combining machine learning and nomogram, guided the improvement of the learning efficiency of the endoscopic assistant, and assisted the construction of an endoscopic assistant system.

## Materials and methods

Fifty patients were randomly selected as the study group when an skilled endoscopic assistant participated in the operation(Group A), and fifty patients were randomly selected as the control group when an unskilled endoscopic assistant participated(Group B). All the randomly selected patients were those who underwent surgical treatment during the same period to ensure that there was no difference in the baseline level of the patients, included patients’ age, sex, preoperative thyroglobulin (TG), preoperative thyroid stimulating hormone (TSH), preoperative antithyroglobulin antibody (TGAb), surgical blood loss, maximum tumor diameter, multifocal tumor, postoperative hoarseness, postoperative hypocalcemia, length of stay, postoperative pathological type, lymphatic metastasis were used to study the total surgical time(TST), the cavity establishment time(CET), the thyroid resection time(TRT), and the lens wipe times(LWT), so as to study the influence of skilled and unskilled endoscopic assistant on the operation and explore the role of endoscopic assistant in the operation. Cumulative Sum(CUSUM) learning curve was used for curve fitting, and the two groups of learning curves were compared. Through univariate and multivariate analysis, the endoscopic assistant influencing factors on the process of surgery was explored. Based on the results of multivariate analysis, a nomogram was constructed to explore the cumulative effect of relevant factors on the surgical process, and a multilayer perceptron (MLP) was used for building a neural network to explore the influence of each relevant factors. All operations were performed by the same surgeon (Flowchart 1). All procedures performed in this study involving human participants were in accordance with the Declaration of Helsinki (as revised in 2013). The study was approved by the Ethic Committee of Sichuan Cancer Hospital(SCCHEC-03–2018-014). Each included patient was interviewed before surgery to inform and obtain their consent, informing them about the purpose, method, subjects’ rights, interests, data protection, and clinical data indicators, operation time, and operation process. They were verbal consented that the included data could be used for the study of endoscopic surgery skills improvement, and their consent was obtained. For the differences in the patients’ educational levels, patient privacy protection, and avoid research leakage, the patients requested not to sign written documents. This study does not involve underage patients.

### Inclusion criteria

(1) Performed the gasless transaxillary endoscopic thyroid lobectomy, (2) Surgery contraindications were excluded, (3) Performed by the same surgeon, (4) The same one skilled and the same unskilled endoscopic assistant.

### Relevant definition

① The skilled endoscopic assistant: Medical personnel who are proficient in endoscopic assistance skills, through skills training, possess surgical qualifications, and have completed more than 100 cases of endoscopic assistant work. ② The sunskilled endoscopic assistant: Medical personnel who have surgical qualifications but have participated in less than 100 endoscopic surgeries. ③ TST: from establishment of the cavity to the complete removal of the thyroid. ④ CET: from skin incision to complete exposure of the thyroid. ⑤ TRT: the time from complete exposure of thyroid to complete removal of the thyroid. ⑥ LWT: The number of times a lens is wiped from the time the skin is cut open to the time the thyroid is completely removed.

### CUSUM analysis

CUSUM values were calculated in the two groups according to the order of operation dates for all cases. CUSUM (1) = the first operative time OT (1)-the mean value OT (mean), CUSUM (n) =OT (n)–OT (mean)+CUSUM (n-1), which was calculated until the CUSUM value of the last case was 0. The cumulative deviation of total surgical time was monitored by CUSUM value as time series accumulated.

### Learning curve fitting

The case number of operation was taken as horizontal coordinate, and the CUSUM value of operation time was taken as vertical coordinate, and the curve was constructed and fitted. When *p* < 0.05 was used to judge the success of curve fitting, R^2^ was used to judge the goodness of fit. The vertex of the CUSUM fitting curve was taken as the minimum cumulative case number of surgeries needed to cross the learning curve, and the learning curve was divided into learning stages and mastery stages.

### Construction a nomogram

Based on multivariate analysis and CUSUM, the nomogram was constructed to predict all relevant factors affecting the duration of surgery, and to evaluate the impact of skilled and unskilled endoscopic assistants on the duration of surgery.

### Multilayer perceptron (MLP) neural network construction

The relevant factors involved in the results of multivariate analysis were included for establishing a neural network model, and the influence of each relevant factors on the duration of surgery were explored. All the data is divided into 7:3 training set and testing set, and fixed random seeds were set to ensure the repeatability of the model. Two hidden layers were set to balance the research purpose and the relevant factors to avoid overfitting, and combined the relationship between the input layer and the output layer to set the number of neurons, and the output results are visually interpreted. The model generated by the training set was used to predict the testing set, and the ROC curve was generated to evaluate the fitting performance of the model, so as to verify the influence of the multivariate analysis results on the duration of surgery with skilled and unskilled endoscopic assistants.

### Statistical analysis

R and SPSS software were used to carry out univariate and multivariate analysis, construct the nomogram and MLP neural network model, and evaluate the model efficiency by ROC curve. The measurement data adopted T test and expressed by (x ± s). Counting data adopted χ 2-test, confidence interval of 95%, with *p* < 0.05 as the difference with statistical significance.

## Results

### Univariate and multivariate analysis

**Figure 1.**
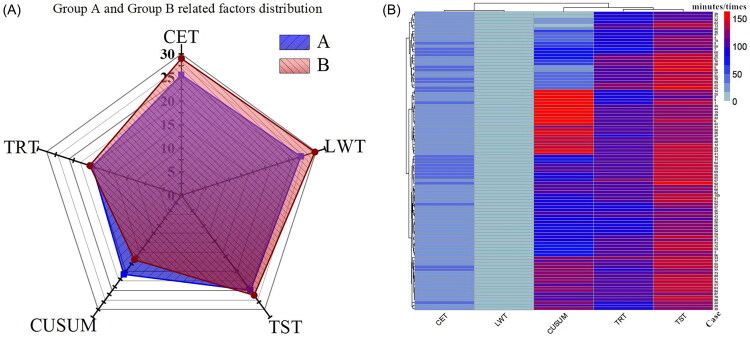
Univariate analysis result and distribution of included relevant factors in each patient. (A)Univariate analysis indicated that CET, TST, LWT and CUSUM were statistically significant. The shorter the time, the smaller the connected area, and the more CUSUM accumulation, the larger the connected area. Even if the LWT and the CET were not much reduced, the TST was also significantly reduced. The lack of overlap in the graph indicates that it still affects the efficiency of the operation. Every minute of endoscopic surgery is very important. (B) Heatmap distribution of all patients on CET, TRT, TST, LWT, CUSUM.

There were no statistical difference in age (*p* = 0.700), sex (*p* = 0.656), preoperative TG (*p* = 0.293), preoperative TSH (*p* = 0.382), and preoperative TGAb (*p* = 0.524), maximum tumor diameter (*p* = 0.840), multifocal tumor (*p* = 0.509), postoperative hoarseness (*p* = 0.695), postoperative hypocalcaemia (*p* = 1.000), length of hospital stay (*p* = 0.486), postoperative pathological type (*p* = 0.771), lymphatic metastasis(*p* = 0.822). (Table 1) The univariate analysis results indicated that there were statistical significance in CET(*p* = 0.001), TST(*p* = 0.001), LWT(*p* = 0.002), CUSUM(*p* = 0.019), but no statistical significance in TRT(*p* = 0.079). However, multivariate analysis indicated that CET(*p* = 0.004), LWT(*p* = 0.013), CUSUM(*p* = 0.025), TRT(*p* = 0.018) had statistical significance (Table 2, Figure 1). Patients with postoperative hoarseness were all difficult airways, and laryngeal mucosal edema was considered after anesthesia intubation.

**Table 1.** Preoperative and postoperative comparison.

		Group A(*n* = 50)	Group B(*n* = 50)	χ^2^/t	P value
Sex(case)	male	15	13	0.198	0.656
	female	35	37		
Age(year)		30.96 ± 6.79	30.44 ± 6.65	0.010	0.700
TSH(mIU/L)		3.63 ± 2.97	4.21 ± 3.55	0.312	0.382
Tg(ng/ml)		34.23 ± 83.12	21.14 ± 27.68	5.130	0.293
TGAb(IU/ml)		37.42 ± 68.53	53.03 ± 158.54	2.350	0.524
Operative blood loss(ml)		8.26 ± 2.49	8.78 ± 3.08	1.040	0.355
Maximum tumor diameter(cm)		12.00 ± 11.13	11.58 ± 9.53	0.578	0.840
Mulifocality(case)	yes	13	16	0.437	0.509
	no	37	34		
Hospital stays(day)		6.54 ± 1.25	6.72 ± 1.33	0.034	0.486
Hypocalcemia(case)	yes	4	4	0.000	1.000
	no	46	46		
Hoarseness after surgery(case)	yes	3	4	0.154	0.695
	no	47	46		
Pathological type	papillocarcinoma	44	46	0.521	0.771
	struma nodosa	4	3		
	Heteromorphic cells	2	1		
lymphatic metastasis(case)	Yes	14	13	0.051	0.822
	No	36	37		

**Table 2.** Univariate and multivariate analysis.

			Univariate analysis		Multivariate analysis		
	Group A	Group B	χ^2^/t	P value	OR(95%CI)		
					Lower	Upper	P value
CET*(minute)	25.58 ± 5.96	29.04 ± 4.36	7.946	0.001	1.042	1.252	0.004
TRT*(minute)	99.02 ± 9.01	102.02 ± 7.83	1.682	P > 0.05	1.012	1.136	0.018
TST*(minute)	124.60 ± 9.36	131.06 ± 8.96	0.785	0.001	NA*	NA*	P > 0.05
LWT*(minute)	9.98 ± 1.61	11.16 ± 2.00	2.020	0.002	1.071	1.799	0.013
CUSUM	103.66 ± 48.73	84.43 ± 29.25	19.813	0.019	0.975	0.998	0.025

* CET: the cavity establishment time; TRT: the thyroid resection time; TST: the total surgical time; LWT: the lens wipe times; NA: Not Available.

* The *p* = 0.166 > 0.05 of Hosmer and lemeshow test indicated that the multivariate analysis had good goodness of fit.

### CUSUM learning curve

**Figure 2.**
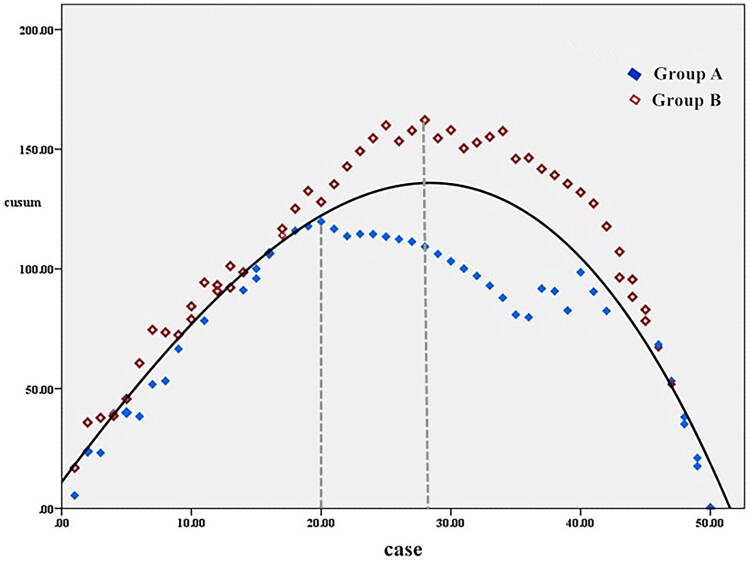
The CUSUM learning curve. The fitting of the learning curve, the cubic equation presented the highest goodness of fit (P < 0.05, with the coefficient of determination R^2^ = 0.807). The fitting equation was: CUSUM(n) = –0.002n³–0.046n^2^+7.251n +10.944 (n represents the surgical case sequence). Group A reached the peak of the curve in 20 case, while group B reached the peak of the curve in 28 case. After that, the fluctuation of group A was smaller than that of group B, and the operation time of group B was close to the peak in 35 case.

Regarding the CUSUM analysis and the learning curve fitting, the cubic equation presented the highest goodness of fit (*p* < 0.05, with the coefficient of determination R^2^ = 0.807). The fitting equation was: CUSUM(n) = −0.002n³–0.046n^2^+7.251n +10.944 (n represents the surgical case sequence). In Group A, the peak was reached when the cumulative number of surgical cases reached 20. In Group B, the highest point when the surgical cases reached 28. The learning stage of Group A was shorter than that of Group B. Moreover, in the subsequent mastery stage, the fluctuations of Group A were smaller than those of Group B. Group B exhibited a significant prolongation of the surgical time at around 35 cases, suggesting that a skilled endoscopic assistant can help the surgery reach a stable operating time as soon as possible while ensuring the stability of the surgical time (Figure 2).

### The nomogram result

**Figure 3.**
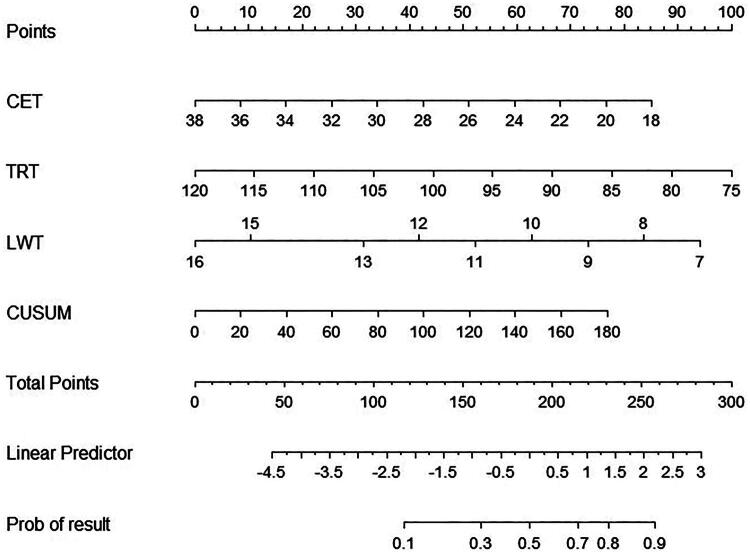
The nomogram result. The nomogram reflects the influence of the accumulation of overall factors on the course of surgical time, suggesting that the shorter the time, the more the score, the more the accumulation of CUSUM, and the more the total score, the greater the probability of improving the surgical efficiency.

With the decrease of time, the corresponding score will gradually increase, and with the increase of time stability, the CUSUM value will also increase, and the corresponding score will gradually increase. When all the scores exceed 300 scores, the possibility of improving the surgical time process will reach the maximum, and the person will be more likely to become an experienced endoscopic assistant (Figure 3).

### The multilayer perceptron result

**Figure 5.**
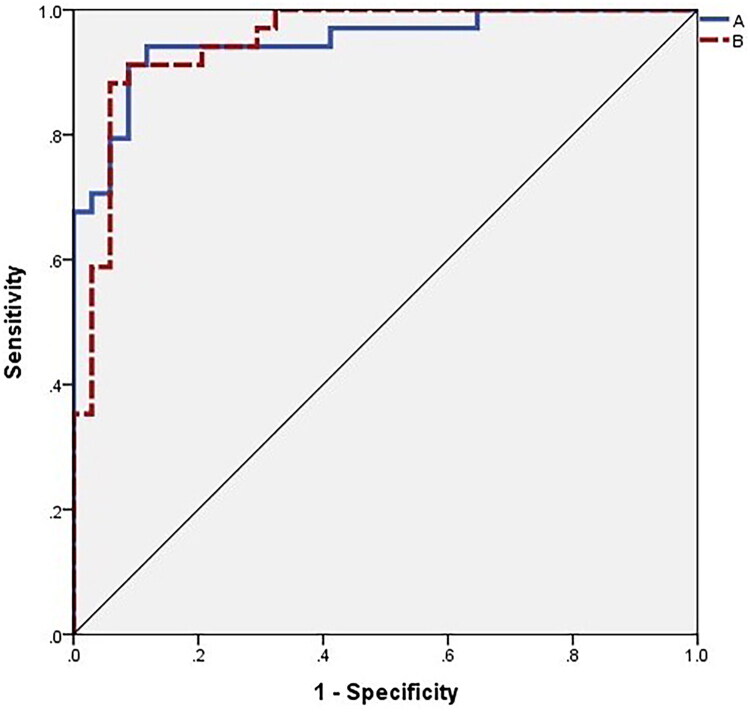
Model predicted ROC curve. The area under ROC curve is 0.949, indicating that the model has a good fit.

The thicker the ‘lines’ in the neural network diagram, the more overlapping the factors, so the stronger the correlation between the corresponding factors and the output results. CUSUM had the greatest effect on the outcome, followed by CET (Figure 4). The neural network model generated by the training set was used to predict the test set, and the area under the ROC curve was 0.949, indicating that the model was well fitted, and the stability of the operation after accumulated experience represented by the CUSUM value was the most influential factor affecting the process of the operation time (Figure 5).

Figure 4.The multilayer perceptron(MLP)result. (A) Neural network diagram: the thicker the lines, the greater the correlation. (B) The influence of each related factors: CUSUM has the greatest influence on the improvement of operation time, which is consistent with the line thickness of the neural network diagram. (C) The fitting prediction interval of the two treatment groups: Group A and group B had a good fit in the interval of 0.8 ∼ 1.0 and 0 ∼ 0.2. (D) The two groups predicted the fitting process: the part where the lines overlapped was the part where the fit was good.

**Figure:**
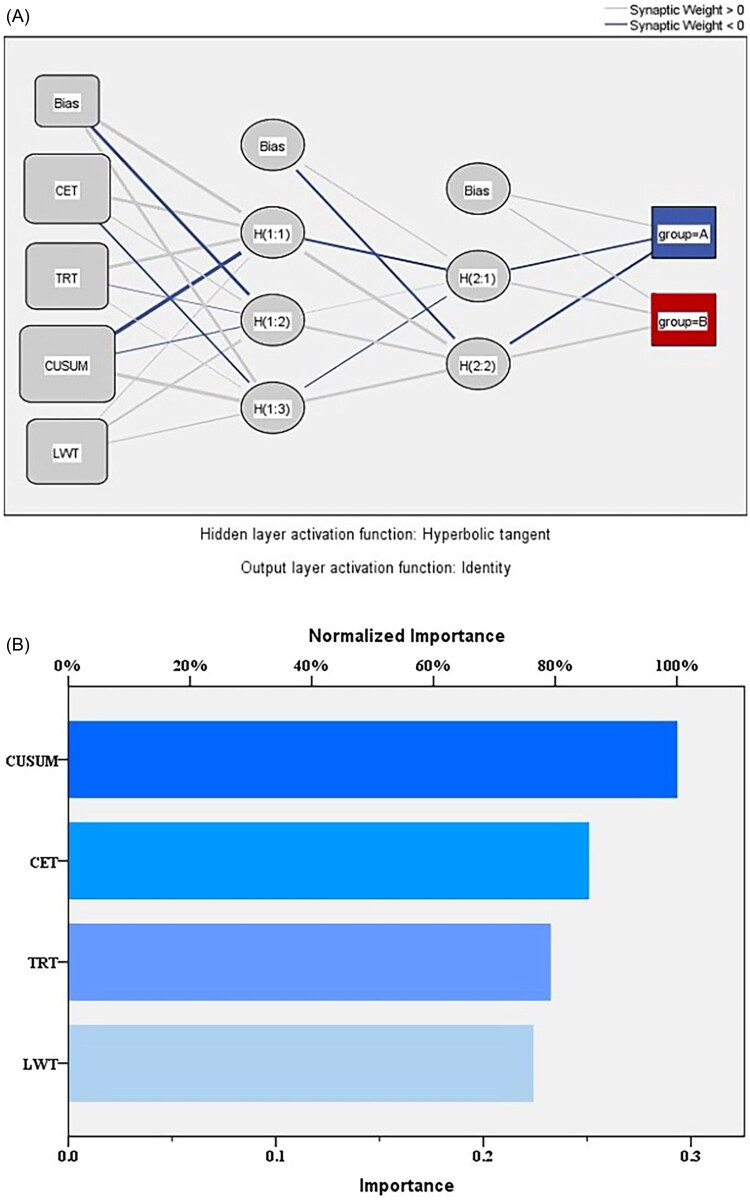

**Figure:**
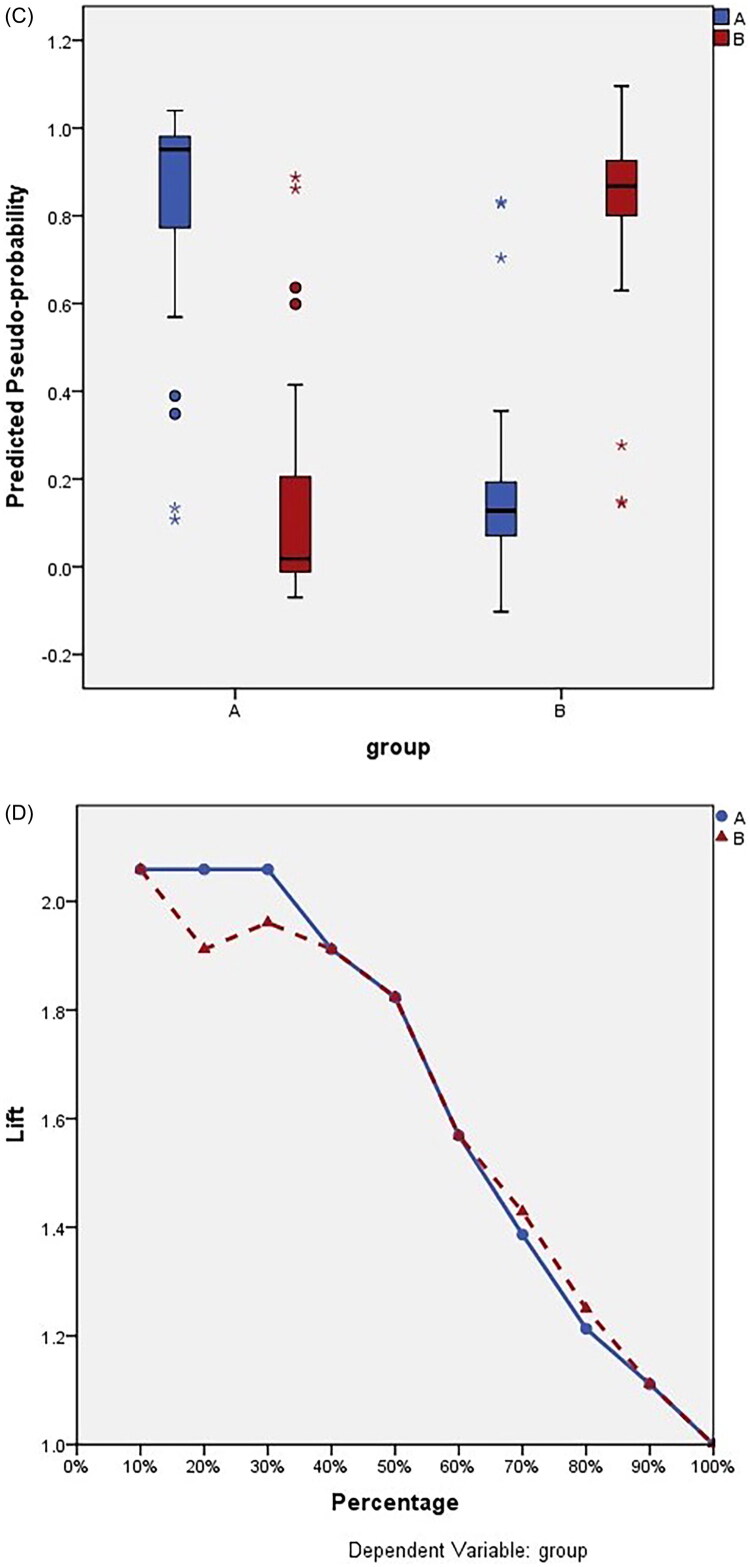

### The endoscopic assistant system establishment scheme

In gasless transaxillary endoscopic thyroidectomy, the skilled endoscopic assistant only spent an average of 4 min less than the unskilled endoscopic assistant during the cavity establishment process, resulting in 7 min less overall operative time. Although the difference was not large, it was statistically significant. Endoscopic surgery requires delicate surgical operations, and every minute is crucial, and the cooperation between the surgeon and the assistant affects the entire endoscopic surgery process in every minute, and the improvement of surgical efficiency is reflected in every minute while taking into account the surgical effect. It is possible to improve the efficiency of surgery even more in the future through active training of endoscopy assistant. Although the number of skilled endoscopic assistants with lens cleaning is only less than 2 times, lens contamination affects the surgical field of view, which also affects the emotion of the surgeon, and also affects the efficiency and safety of surgery. Compared with operations involving unskilled endoscopic assistants, operations involving skilled endoscopic assistants can help shorten CET, effectively assist thyroidectomy, reduce LWT, and effectively improve surgical efficiency. In addition, the learning curve of the skilled endoscopy assistant led to faster surgical time reaching the mastery stage and less fluctuation in the operative time of the mastery stage. It is necessary to conduct endoscopic assistant training. So how can we improve? The following is to explore the establishment of endoscopic assistant system.

#### Preparation before the surgical procedure

**Figure 6.**
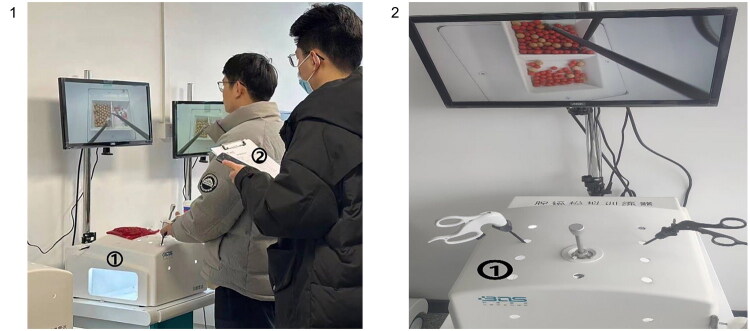
Preclinical training. Operation training before the surgical process: ① Endoscopic training instruments: assistants operate in the simulated space with endoscopic instruments, and the screen mimics the perspective of the endoscopic lens for excision, suture and other operations; ② trainers conduct training scores; Based on the nomogram and machine learning, in order to reduce CET and TRT time, preclinical training should strengthen the coordination of lens stability training during separation and the stability training of clamping exercises, and practice the ability of lens blur processing to reduce LWT.

In gasless transaxillary endoscopic thyroidectomy, where the surgical angle of view is quite different from that of traditional surgery, the assistant should practice the basic surgical operations under the endoscopic field of view, such as knotting. Before entering the surgical procedure, the endoscopic assistants should use the endoscopic training instrument for practice (Figure 6). Based on the nomogram and machine learning, in order to reduce CET and TRT time, preclinical training should strengthen the coordination of lens stability training during separation and the stability training of clamping exercises, and practice the ability of lens blur processing to reduce LWT. In addition, the assistants need read the patient’s CT imaging. Before the operation, the assistant should learn the adjustment method of the lens, how to adjust the focal length, resolution, and so on.

#### Assisted operation during surgery

Share the experience of endoscopic assistant according to surgical procedure (Flowchart 2).

**Flowchart 1.**
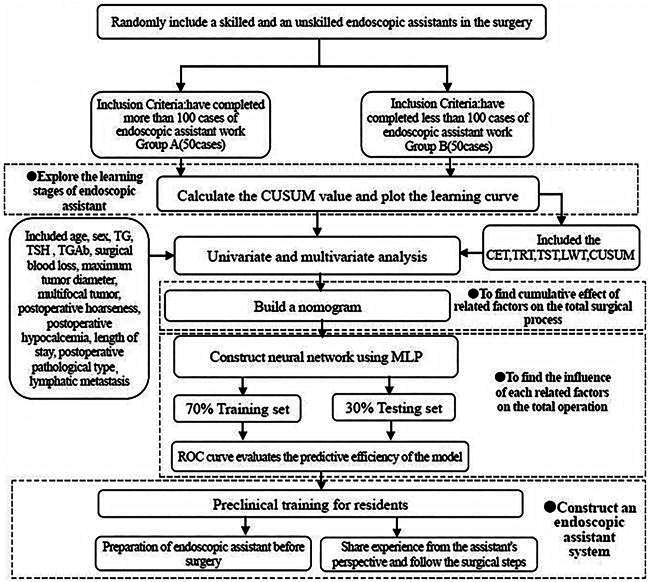
Research flowchart. CET: the cavity establishment time; TRT: the thyroid resection time; LWT: the lens wipe times; TST: the total surgical time; MLP: Multilayer Perceptron; CUSUM: Cumulative Sum.

**Flowchart 2.**
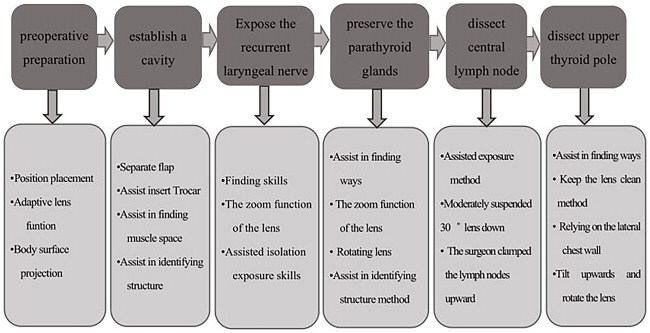
Share the experience of endoscopic assistant according to the surgical procedure from the perspective of endoscopic assistant.

## Discussion

With the rise in physical examinations, the incidence of thyroid diseases has been climbing annually. Thyroidectomy has emerged as a crucial treatment method for thyroid diseases, particularly for papillary thyroid microcarcinoma that necessitates only the removal of a thyroid gland lobe. The variety of surgical methods has garnered considerable interest [13]. Hence, surgeons are exploring techniques that maintain the efficacy of traditional surgery while also prioritizing aesthetics and safety. Endoscopy and its associated robotic surgical procedures have emerged as prominent choices for minimally invasive thyroidectomy [14–20]. Currently, endoscopic thyroidectomy primarily encompasses axillary, areolar, and oral approaches, though their indications remain a subject of debate. The American Thyroid Association (ATA) and The Chinese Thyroid Association(CTA) have put forth relative indications considering aspects like age, tumor size [21,22]. The age criteria range from 15∼45 years, accommodating benign tumors ≤ 6 cm or malignant tumors ≤ 2 cm, without no extensive. The oral approach, which leverages a natural cavity, is contentious. It mandates patients to exhibit standard thyroid function, precise neck anatomy, no oral diseases, and other prerequisites. Additionally, changing the original thyroidectomy incision method introduces risks such as oral infections. The areolar method requires CO_2_ injection to establish the cavity, potentially causing hypercapnia, subcutaneous emphysema, and elevated intracranial pressure [23–27]. Therefore, given the operational space and the learning curve, gasless transaxillary endoscopic thyroidectomy stands as an ideal choice for novices.

The report on the experience of endoscopic assistants primarily centers around thoracoscopic and laparoscopic surgeries, while limited documentation on assistants for thyroid endoscopy. Nonetheless, endoscopic thyroidectomy demands a superior level of assistance due to the natural divide between the chest and abdominal cavity. Endoscopic assistants play a crucial role right from the initiation of the cavity establishment, given the constrained space after its formation. As a result, they must exercise flexible judgment in accordance with the surgeon’s needs. The endoscopic assistant is often likened to the ‘eye’, a role they similarly fulfill in thyroid endoscopic surgeries [28,29]. The development of endoscopic assistants is intertwined with their rapport with surgeon, and a surgeon’s surgical habits can influence the experience acquisition of assistants [30]. Zhen et al. [31] analyzed the clinical data of 118 patients who underwent endoscopic thyroidectomy. They concluded that the assistant should sustain a ‘Y’ shaped tunnel on display and clear the neck area, They should use the sternocleidomastoid muscle as a reference, maintain an isosceles triangle on the monitor, with the thyroid cartilage level forming the base and the medial edge of the sternocleidomastoid muscle on both sides serving as the sloping edges. The study underscored the pivotal nature of the endoscopic assistant’s active collaboration during the surgery.

Although we had attempted a larger sample size when setting up the groups, we found that when the cases accumulated to a certain degree, the cooperation between the surgeon and the assistant became very harmonious and the operation time tends to be stable. The main factors affecting the surgical process were the patient’s condition and some other factors. At the same time, we also used 50 cases as the standard for evaluating the endoscopic assistant ability. However, our research concludes that the establishment of the endoscopic assistant system is necessary. It can not only shorten the operation time, but also reduce the duration of anesthesia, protect endoscopic instruments, stabilize the emotions of the surgeon, improve the ability of beginners in endoscopic thyroidectomy, and even promote the endoscopic assistant to become the surgeon as soon as possible.

Our research compensates for the influence of endoscopic thyroid assistants on the duration of surgery and emphasizes the importance of endoscopic assistants in gasless transaxillary endoscopic thyroidectomy. we evaluated the influence of endoscopic assistant on the duration of surgery at different stages by fitting the learning curve. By calculating the CUSUM value, we could carry out the influence of accumulated operation time on the duration of surgery. Through multivariate analysis, CUSUM was included to find the related factors of the influence of endoscopic assistant on the duration of surgery. The cumulative influence of all related factors on the operation time was predicted by constructing a nomogram, and the influence of each factor on the operation time was evaluated by MLP.

MLP is a deep learning model composed of three parts: input layer, one or more hidden layers, and output layer. It can find the influence of related factors on the linearity and nonlinearity of the outcome, and balance the weight through the characteristics of forward and backward propagation to reduce the difference between the predicted value and the actual value. We evaluated the influence of each relevant factors on the duration of surgery through MLP, and found that CUSUM became the most influential factor, so the accumulation of experience of endoscopic assistant became an important factor to improve the learning efficiency. Similarly, Li [32] built a prediction model of thyroid lymph node metastasis based on MLP, and considered the influence of MLP on each factors with better visualization. However, MLP has overfitting, which may amplify the influence of some factors. We first screen the relevant factors through multivariate analysis, and then use it to coordinate the relationship between the sample size and the research factors to avoid overfitting caused by generalization. Meanwhile, the overfitting was reduced by attempting to set the hidden layer and the neuron. Finally, the evaluation of the overall effect of related factors through nomogram can balance the bias of the individual effects of each factor.

Meanwhile, we considered CET, LWT, TST and CUSUM as the main factors affecting the surgical process through univariate analysis result, but multivariate analysis suggested that TRT replaced TST time as the main factors affecting surgical process. The reason why we set up TST time was actually the result of the addition of CET and TRT. Therefore, considering that the interaction of internal factors will affect the multivariate results, TRT had a greater impact on the multivariate results.

Based on the influence of the accumulation of experience of the endoscopic assistant on the surgical process, we put forward the concept of establishing the endoscopic assistant system, seeking to establish the endoscopic assistant system through preclinical training, preoperative preparation, and intraoperative experience sharing through the perspective of the endoscopic assistant. Several institutions worldwide have recommended training modalities for laparoscopic surgical assistants. The process begins with a laparoscopic simulator for training, followed by three-dimensional spatial operation training in a two-dimensional setting. Subsequently, animal experiment training offers a sense of spatial positioning under the endoscope, culminating in clinical practice. Li [33] outlined foundational assistance techniques for endoscopic assistants, emphasizing lens understanding, particularly discerning between 0°and 30° lenses, and mastering lens orientation, aperture adjustments, and zoom functionalities. Chang [34] proposed that endoscopic assistants should reflect after each surgery, which includes reviewing surgical footage, facilitating the enhancement of their surgical assistance expertise.

Our research focuses on the endoscopic assistant should adhere to the principle of ‘the lens follows the knife,’ adjusting the field of view based on the situation. During delicate or critical operations, a close-range field of view is preferable to enable the surgeon to discern vascular and neural anatomical relationships, such as when dealing with the inferior thyroid artery or separating the recurrent laryngeal nerve. For identification of organ structures or anatomical positions, a distant field of view is appropriate, for instance, when establishing a cavity tunnel. Presurgical endoscopic preparation is crucial. It’s also vital to acquaint oneself with lens specifications prior to surgery, such as focal length adjustment and aperture scaling, since lens functionalities might differ.

In addition, there are many limitations in our research. The preoperative inclusion factors for patients were limited, and factors such as patient weight. Multicenter randomized controlled study need to be set up. In addition, the other postoperative complications were not included, and there was a lack of long-term follow-up process.

## Conclusion

In gasless transaxillary endoscopic thyroidectomy, skilled endoscopic assistants can reduce the LWT times, reduce the CET, TRT, effectively shorten the surgical process and the accumulation of surgical experience is helpful to improve surgical efficiency. The establishment method should be carried out from preclinical training, preoperative preparation, and accumulate surgical operation experience.

## Data Availability

The datasets used and/or analyzed during the current study are available from the corresponding author upon reasonable request.
